# Subduralhämatom und Pneumatozephalus nach neuroaxialer Anästhesie: Abklärung von postpunktionellem Kopfschmerz nach geburtshilflicher Periduralanästhesie

**DOI:** 10.1007/s00101-021-01077-5

**Published:** 2021-12-18

**Authors:** Kateryna Kovalevska, Rüdiger Hochstätter, Michael Augustin, Gregor Alexander Schittek, Helmar Bornemann-Cimenti

**Affiliations:** 1 Klin. Abt. für allgemeine Anästhesiologie, Notfall- und Intensivmedizin, Medizinische Universität Graz, Auenbruggerplatz 5/5, 8036 Graz, Österreich; 2 Klin. Abt. für Geburtshilfe, Universitätsklinik für Frauenheilkunde und Geburtshilfe, Medizinische Universität Graz, Graz, Österreich; 3 Universitätsklinik für Radiologie, Medizinische Universität Graz, Graz, Österreich

## Einleitung

Der Wunsch einer Gebärenden nach Schmerzlinderung stellt, unabhängig vom Muttermundsbefund während der Eröffnungsphase, eine Indikation zur lumbalen Periduralanästhesie (PDA) dar; diese ist ein etabliertes Verfahren zur Linderung der Wehenschmerzen während der Geburt [1]. Je nach Erfahrung des Durchführenden treten akzidentelle Duraperforationen (ADP) bei geburtshilflicher PDA-Anlage in bis zu 3,6 % der Fälle auf [2]. Der postpunktionelle Kopfschmerz („postdural puncture headache“, PDPH) ist eine mögliche Folge der ADP. Die Inzidenz von PDPH nach einer ADP mit einer Touhy-Nadel größeren Kalibers (16-18 G) beträgt bis zu 50 % [5].

Dem PDPH kommt ein besonderer Krankheitswert zu, weil die betroffenen jungen Frauen neben dem persönlichen Leidensdruck auch ihr neugeborenes Kind nur eingeschränkt versorgen können [1]. Zudem bestehen Hinweise darauf, dass der PDPH die Entstehung chronischer Kopfschmerzen begünstigt [4].

Differenzialdiagnostisch sollten bei Patientinnen, die nach einer PDA-Anlage (mit oder ohne ADP) Kopfschmerzen entwickeln, das Vorliegen von Migräneanfällen, einer Spätform einer Präeklampsie, von Spannungskopfschmerzen, Subduralhämatomen (SDH), Meningitiden oder einer zerebralen Sinusvenenthrombose in Erwägung gezogen werden [2, 6]. In diesem Zusammenhang wurde 2014 erstmals ein Fall mit einer Kombination aus Pneumatozephalus und subarachnoidaler Hämorrhagie beschrieben [11]. Im Fallbericht war eine 32-jährige Patientin 3 Tage nach einer PDA-unterstützten Entbindung mit starken Kopfschmerzen vorstellig geworden. Im weiteren Verlauf entwickelte sie aufgrund von intrakraniellen Blutungen und Lufteinschlüssen tonisch-klonische Krämpfe, die eine 29-tägige intensivmedizinische Behandlung notwendig machten. Im vorliegenden Beitrag wird ein ähnlicher Fall einer Patientin präsentiert; diese wies ebenso intrakranielle Luft und Blutungen nach einer PDA auf, allerdings stellte sich im Rahmen einer interventionellen Behandlung ein deutlich besserer Verlauf ein.

## Fallbericht

Eine 20-jährige Primigravida ohne Vorerkrankungen hatte initial in der 40 + 1 SSW in einem peripheren Krankenhaus der Grundversorgung komplikationslos nach Anlage einer PDA vaginal entbunden. Sie stellte sich am 6. postpartalen Tag mit orthostatischen Kopfschmerzen, leichter Übelkeit ohne Erbrechen und schmerzbedingt eingeschränkter Leistungsfähigkeit in der geburtshilflichen Ambulanz der Medizinischen Universität Graz vor. Die Patientin berichtete, dass die Schmerzen seit dem 4. postpartalen Tag anhielten.

In der initialen Untersuchung zeigten sich keine neurologischen Defizite und kein Meningismus. Im lokalen Befund war die Einstichstelle nicht mehr eindeutig identifizierbar; es bestand kein Hinweis auf eine Infektion bzw. eine Druckdolenz. Der Allgemeinstatus der Patientin war, bis auf die leicht erhöhten Entzündungszeichen unauffällig (37,7 °C, Herzfrequenz 73/min, Blutdruck 125/78 mm Hg, Body-Mass-Index [BMI] 29,6 kg/m^2^, Leukozytenzahl 12.000/µl, C‑reaktives Protein [CRP] 12,4 mg/l, restliche Laboruntersuchungen ohne pathologische Befunde, Gerinnungsanamnese unauffällig). Lediglich eine leichte Übelkeit gab die Patientin noch an.

In der externen Anästhesiedokumentation war die PDA-Anlage mit 2 Punktionsversuchen auf der Höhe L2–L3 nach einem frustranen Versuch auf der Höhe L3–L4 mithilfe einer 18-G-Tuohy-Nadel protokolliert. Ob Luft oder 0,9 %ige NaCl-Lösung zum Aufsuchen des Epiduralraums appliziert wurde, war nicht vermerkt worden. Es wurde jedoch dokumentiert, dass kein Blut oder Liquor aspiriert werden konnte. Der Katheter wurde nach der Applikation einer Testdosis fixiert. Unmittelbar nach der Anlage sowie peripartal bis zur Entfernung des PDK wies die Patientin keine Auffälligkeiten auf.

Aufgrund dieser Gesamtkonstellation entschieden sich die diensthabenden Gynäkologen für die Einbindung der Neurochirurgen. In der Untersuchung durch den hinzugezogenen Neurochirurgen zeigte sich die wache, orientierte Patientin mit isokoren, mittelweiten, lichtreagiblen Pupillen ohne Nystagmus oder okulomotorische Auffälligkeiten. Es ergab sich kein Hinweis auf Hirnnervenausfälle, und die Sprache der Patientin war unauffällig. Sie bot keinen Anhalt für sensomotorische Defizite oder Pyramidenbahnzeichen. Die Intensität der Kopfschmerzen im Stehen wurde auf einer numerischen Rating-Skala (NRS) von 0 bis 10 bei 6–7 eingestuft, im Liegen linderten sich die Schmerzen bis zur Stufe 2.

**Figure 1:**
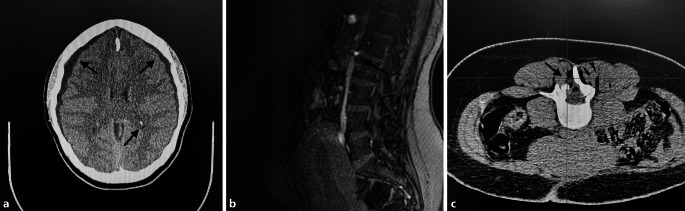

Aufgrund der verzögerten klinischen Zeichen und des erheblichen Leidensdrucks der Patienten empfahl der Neurochirurg eine Untersuchung mithilfe der kranialen Computertomographie (cCT) in domo. In dieser stellten sich ein 7 mm breites, rechtseitiges, nichtfrisches SDH sowie ein 5 mm breites, linksseitiges, nichtfrisches SDH dar. Zusätzlich fanden sich Lufteinschlüsse im Bereich der Hinterhörner und im Vorderhorn des rechten Seitenventrikels (Abb. 1a).

Daraufhin erfolgten die stationäre Aufnahme der Patientin und die Einleitung einer medikamentösen Therapie mit Paracetamol, 1 g i.v. 4‑mal tägl., und Koffein, 200 mg p.o. 3‑mal tägl., gemäß den aktuellen Empfehlungen [2, 3]. Der neurochirurgischen Empfehlung folgend wurde eine weiterführende Diagnostik mithilfe der Magnetresonanztomographie (MRT) geplant und die medikamentöse Therapie nach Reevaluierung um Theophyllin, 250 mg i.v. 3‑mal tägl., ergänzt.

Das am Folgetag durchgeführte MRT der Lendenwirbelsäule (LWS) zeigte auf Höhe L2 eine Flüssigkeitsanlagerung, vereinbar mit dem Zugangsweg der PDA (Abb. 1b). Die Neurochirurgen in domo sprachen ihre Empfehlung zur CT-gestützten Applikation eines epiduralen Blutpatch (EBP) aus. Dieser Vorschlag wurde am nächsten Tag in domo durch die interventionellen Radiologen umgesetzt (Abb. 1c). Der Zugang wurde auf Höhe L3/L4 gewählt. Unter sterilen Bedingungen sollten 20 ml Eigenblut appliziert werden. Die Punktion wurde jedoch nach der Injektion von 9 ml abgebrochen, weil die Patientin über Parästhesien in der linken unteren Extremität klagte.

Die Patientin verblieb noch einen Tag stationär zur postinterventionellen Observanz. Diese verlief ohne Zwischenfall, und die Patientin konnte am Folgetag nach deutlicher Beschwerdelinderung (NRS 2–3, keine Übelkeit/Erbrechen) entlassen werden konnte. Nach einer Woche (16. Tag postpartum) wurde die Patientin telefonisch kontaktiert. In dem Telefonat berichtete sie über die vollständige Remission, sodass auf weitere Diagnostik und bildgebende Untersuchungen verzichtet werden konnte.

## Diskussion

Ein SDH ist eine sehr seltene, aber schwerwiegende Komplikation nach einer PDA bzw. Spinalanästhesie [5]. Speziell in der Geburtshilfe hat diese Komplikation direkten Einfluss auf die Versorgung des Neugeborenen durch die Mutter und führt möglicherweise bei Folgeschwangerschaften zu einer ablehnenden Haltung der Betroffenen gegenüber der geburtshilflicher Anästhesieverfahren [4]. Das SDH stellt die Folge einer venösen Sickerblutung aus Brückenvenen zwischen der Dura und der Arachnoidea dar. Dies erklärt sich durch ein punktionsbedingtes Leck der Dura mit Austritt von zerebrospinaler Flüssigkeit („cerebrospinal fluid“, CSF). Ein kontinuierlicher CSF-Verlust verursacht den Abfall des intraspinalen und intrakranialen Drucks.

Differenzialdiagnostisch sollte bei persistierenden Kopfschmerzen trotz adäquater konservativer Therapie und evtl. appliziertem EBP durch einen Anästhesisten immer ein SDH in Erwägung gezogen werden; dieses lässt sich mithilfe der CT- oder MRT-Diagnostik bildmorphologisch nachweisen.

Im Rahmen des neuroaxialen Anästhesieverfahrens sind 2 Hauptmechanismen für die Entwicklung eines Pneumatozephalus beschrieben. Die „Ball-valve“-Theorie besagt, dass Niesen, Husten, Valsalva-Manöver und andere Ereignisse mit Bildung erhöhter positiver Druckspitzen die Luft durch den duralen Defekt drücken, diese allerdings danach nicht mehr entweichen kann, was zur Luftretention führt. Bei größeren Luftmengen kann auf diesem Weg ein Spannungspneumatozephalus entstehen. Nach der „Inverted-bottle“-Theorie führt eine Drainage der CSF zu einem negativen intrakraniellen Druckgradienten, der den Zustrom von Luft ermöglichen kann [6]. Außerdem wird für die Identifikation des Epiduralraums mithilfe der „Loss-of-resistance“-Technik teilweise nach wie vor eine luftgefüllte Spritze eingesetzt. Bei der Verwendung von Luft vs. Kochsalzlösung ist die Inzidenz von PDPH signifikant erhöht, außerdem kommt es häufiger zur inkompletten Ausbreitung und schlechter Analgesie [7–9].

Ein EBP hat mit Erfolgsraten zwischen 50 und 80 % eine hohe Effektivität bei der kausalen Behandlung von PDPH [6, 7]. Somit wird der EBP als Methode der Wahl empfohlen. Praktisch wird dieser allerdings häufig ohne eine bildgebende Unterstützung appliziert. Aufgrund des verzögerten Auftretens der Kopfschmerzen hatten sich die Behandelnden in der geburtshilflichen Ambulanz bei dieser Patientin für die Einbindung der Neurochirurgen entschieden, die wiederum ein CT-gestütztes Vorgehen indizierten, um das Risiko einer weiteren ADP zu minimieren und zugleich die Ausbreitung des Blutes in den relevanten Segmenten zu verifizieren.

Viele Patientinnen haben möglicherweise Angst vor neuerlichen neuroaxialen Interventionen. Außerdem ist der EBP selbst ein komplikationsreiches Verfahren. Deshalb sollen auch andere nichtinvasive Optionen in das Behandlungskonzept, wie z. B. eine intranasale Lidocainvernebelung, eingeschlossen werden [10].

## Fazit für die Praxis

- Subduralhämatome und Pneumatozephalus gehören zu den seltenen und potenziell lebensbedrohlichen Komplikationen neuroaxialer Anästhesieverfahren. Pathophysiologisch können sich diese sowohl durch Zug auf die Meningen bei Liquorverlust als auch spontan durch Pressen bei der Wehentätigkeit entwickeln.
- Ziel des Fallberichts ist eine Vigilanzerhöhung bezüglich dieser Komplikationen, die immer differenzialdiagnostisch in Erwägung gezogen werden müssen, v. a. bei Auftreten von therapieresistentem postpunktionellem Kopfschmerz („postdural puncture headache“, PDPH). Zusätzlich soll auf die Möglichkeit hingewiesen werden, einen epiduralen Blutpatch (EBP) durch versierte Interventionisten CT-gestützt applizieren zu lassen.
- Die Verwendung von Luft zur Identifikation des Epiduralraums bei der Anlage der Epiduralkatheter kann zur Verminderung des analgetischen Effekts, zu häufigerem Auftreten von PDPH und zu höheren Raten der akzidentellen Duraperforationen führen.
- Die primäre CT-gesteuerte Applikation eines EBP bietet hinsichtlich der Genauigkeit und Prophylaxe erneuter duraler Perforationen einen Sicherheitsvorteil. Allerdings sollten auch nichtinvasive Therapiemethoden in Erwägung gezogen werden.
